# A spectrum of sharing: maximization of information content for brain imaging data

**DOI:** 10.1186/s13742-014-0042-5

**Published:** 2015-01-29

**Authors:** Vince D Calhoun

**Affiliations:** The Mind Research Network & LBERI, 1101 Yale Blvd NE, Albuquerque, New Mexico 87106 USA; Department of ECE, University of New Mexico, Albuquerque, New Mexico USA

**Keywords:** Data sharing, Privacy, Classification, Multivariate, Neuroinformatics, Deep learning, Independent component analysis

## Abstract

Efforts to expand sharing of neuroimaging data have been growing exponentially in recent years. There are several different types of data sharing which can be considered to fall along a spectrum, ranging from simpler and less informative to more complex and more informative. In this paper we consider this spectrum for three domains: data capture, data density, and data analysis. Here the focus is on the right end of the spectrum, that is, how to maximize the information content while addressing the challenges. A summary of associated challenges of and possible solutions is presented in this review and includes: 1) a discussion of tools to monitor quality of data as it is collected and encourage adoption of data mapping standards; 2) sharing of time-series data (not just summary maps or regions); and 3) the use of analytic approaches which maximize sharing potential as much as possible. Examples of existing solutions for each of these points, which we developed in our lab, are also discussed including the use of a comprehensive beginning-to-end neuroinformatics platform and the use of flexible analytic approaches, such as independent component analysis and multivariate classification approaches, such as deep learning.

## Review

Wide-spread sharing of neuroimaging data and results is gaining momentum despite initial bold attempts which failed to gain widespread adoption initially [1,2]. Recently, calls for neuroimaging data sharing have been revived [3], though there is a lack of consensus about ideal models for incentivizing data sharing. Indeed there are many issues to consider, such as when to best consider sharing (e.g., at study setup, or after study completion), incentives to both data providers and data consumers, resources and sustainability, type of data to be shared (e.g., summary results or raw data), as well as the use of analytic approaches ranging from a high-level summary (e.g., meta-analytic) to data-driven and multivariate approaches.

**Figure 1:**
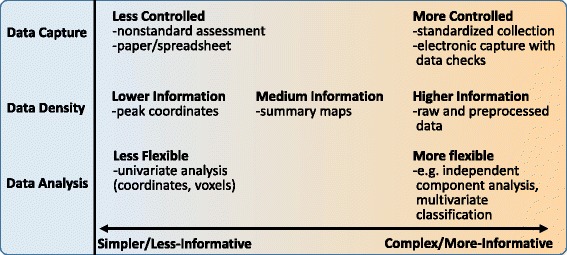
**A spectrum of data-sharing.**

Data sharing involves balancing many trade-offs. In this paper, we consider the larger issue of data sharing as seen through the lens of a spectrum ranging from simpler and less informative, to more complex and more informative. We consider, one-by-one in the subsequent sections, three domains within this context including data capture, data density, and data analysis (Figure 1) with a primary focus on how to push towards the right end of the spectrum to maximize information content while addressing existing challenges.

First, regarding data capture, it almost goes without saying that it makes sense to maximize the quality of data as it is collected. However, most studies still do not collect data fully electronically, do not store all aspects of study information in a central place, and still draw upon error prone data entry and use of individual spreadsheets. In addition, one of the most difficult aspects of sharing data across studies relates to mapping the assessment information into a common framework. Indeed, the development of common data elements for various domains is the focus of much research [4-6]. Secondly, the type of data shared ranges from higher to lower information density from time series data (e.g., an fMRI data set), to contrast or connectivity maps, to coordinates and peaks (e.g., tables in a journal article). And finally, the flexibility of the analytic approach is tightly tied into the availability of data at hand and also directly related to the amount of information one can extract from the data. We discuss all three of these issues and make some recommendations which we hope will be useful for the field.

### Data capture

**Figure 2:**
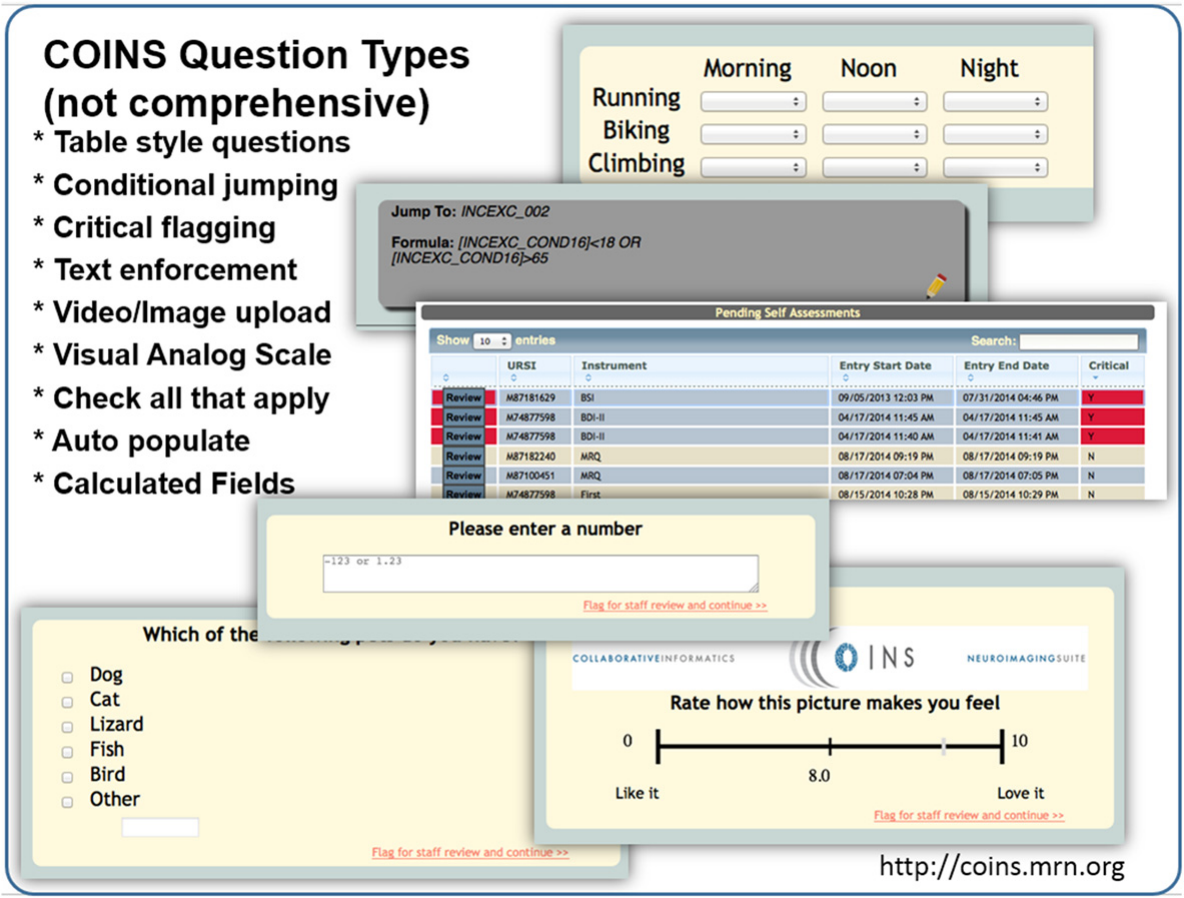
**Examples of question types included in COINS.**

One of the perhaps more overlooked aspects of data sharing involves the data collection phase. Most studies are still focused primarily on optimizing the data collection process within the study despite considerable evidence that a more comprehensive approach reduces errors [7], and sharing of the data is seen as a secondary phase or perhaps relegated to a ‘necessary burden’ [8]. As seen in Figure 1, the data collection process for assessments can benefit greatly from the use of electronic capture tools such as redcap [9], but there is a notable paucity of tools which can handle both advanced assessments, as well as neuroimaging (or genetics) data. One such tool we have developed is the collaborative informatics and neuroimaging suite (COINS) [10]. COINS provides a multitude of tools to maximize the efficiency and minimize the errors associated with collecting both assessment and neuroimaging data [11,12], and also provides tools to easily enable the sharing of data from within this framework [13]. Tools such as those in COINS are important not only to collect quality data, but also to encourage adoption of common data elements that enable mapping of assessment and clinical information in addition to imaging information between studies [4-6,14] (see Figure 2).

#### Concern: Storage

**Figure 3:**
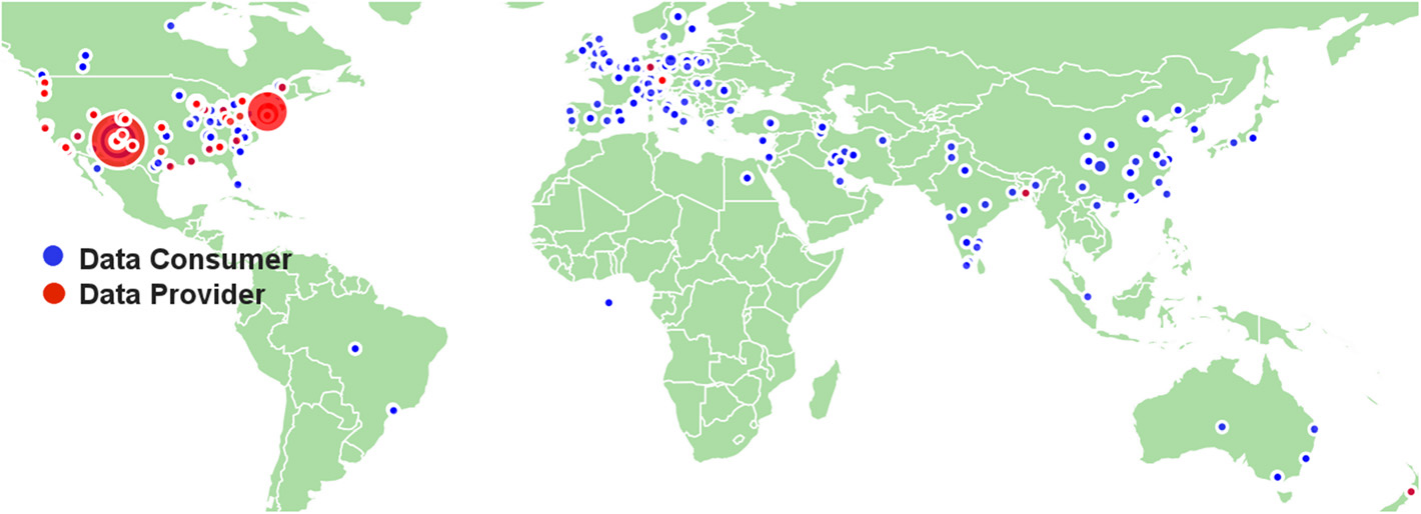
**Map of COINS data distribution (consumers and providers).**

One of the concerns relevant to neuroimaging data (and becoming even more so with the increased pace of data collection [15]) is the amount of data storage required to store the time series data. Storage can be addressed in multiple ways including the use of cloud-based storage [16], the centralization of large-data capacity, and the use of distributed approaches [17]. While database management is a long-standing topic of discussion, the relatively small sample sizes used in most imaging studies [18], combined with a research silo culture (i.e., working within a lab and not sharing information across labs), have left most researchers with little incentive to invest in developing and/or adopting sophisticated databases. Recent changes include an increasing number of multisite studies [19,20], release of data from open science initiatives [21], and the expansion of imaging and phenotypic data acquisition protocols (e.g., the introduction of multiband imaging [22] have produced a nearly 40-fold increase in the size of functional and diffusion datasets). Multiple neuroinformatics tools are emerging to facilitate data organization and sharing, including XNAT [23], LONI [24], and BIRN HID [25] – each of which, is a work in progress with unique advantages and disadvantages, as well as uncertain readiness for widespread deployment. At the Mind Research Network (MRN), we have developed COINS, a sophisticated system for study management, archiving, and sharing; it currently serves multiple investigators and imaging centers around the world [11]. COINS can handle a variety of imaging modalities and analysis tools, as well as data capture and archival services that automate the transfer, organization, backup and processing of imaging data directly from the MRI scanner. For collecting phenotypic data, COINS provides an easy-to-use form builder that generates questionnaires of varying complexity for web-based data entry, for use by participants at home or in a research office. A tool called oCOINS (offline COINS) facilitates offline data entry for fieldwork; it features the necessary synchronization and security features (e.g., differential role and permission setting). COINS’ data collection and organization features are complemented by a graphical “Data Exchange” tool which enables searching, identification, and sharing of datasets between users (or others, with permission) [11,12]. Containing over 550 studies, 37,000+ imaging sessions from 30,000+ subjects and 395,000+ assessments, COINS has undergone substantial testing and continues to rapidly grow [13,26]. A map of the locations where data has been provided or downloaded is provided in Figure 3. Based on the large amount of download activity (and this is not a unique phenomenon to COINS), it is clear there is a great demand for more open data sharing in the neuroimaging community.

In summary, the use of standardized tools for capturing and organizing data, is essential as they have been shown to both reduce errors, as well as increase efficiency of data capture [27-29]. There are many tools available for capturing assessment data [29-32], though such solutions are not used as much as they should be in neuroimaging studies, especially for neuroimaging data, and the studies that do tend to use separate systems for neuroimaging and assessment data. However there are some notable exceptions to this and a large growth in the number of neuroinformatics tools available to the community. The community will benefit greatly from an increase in integrated systems where querying for multiple data types (e.g., neuroimaging, assessment, genetics, social media) is possible via a single entry point.

### Data density

**Figure 4:**
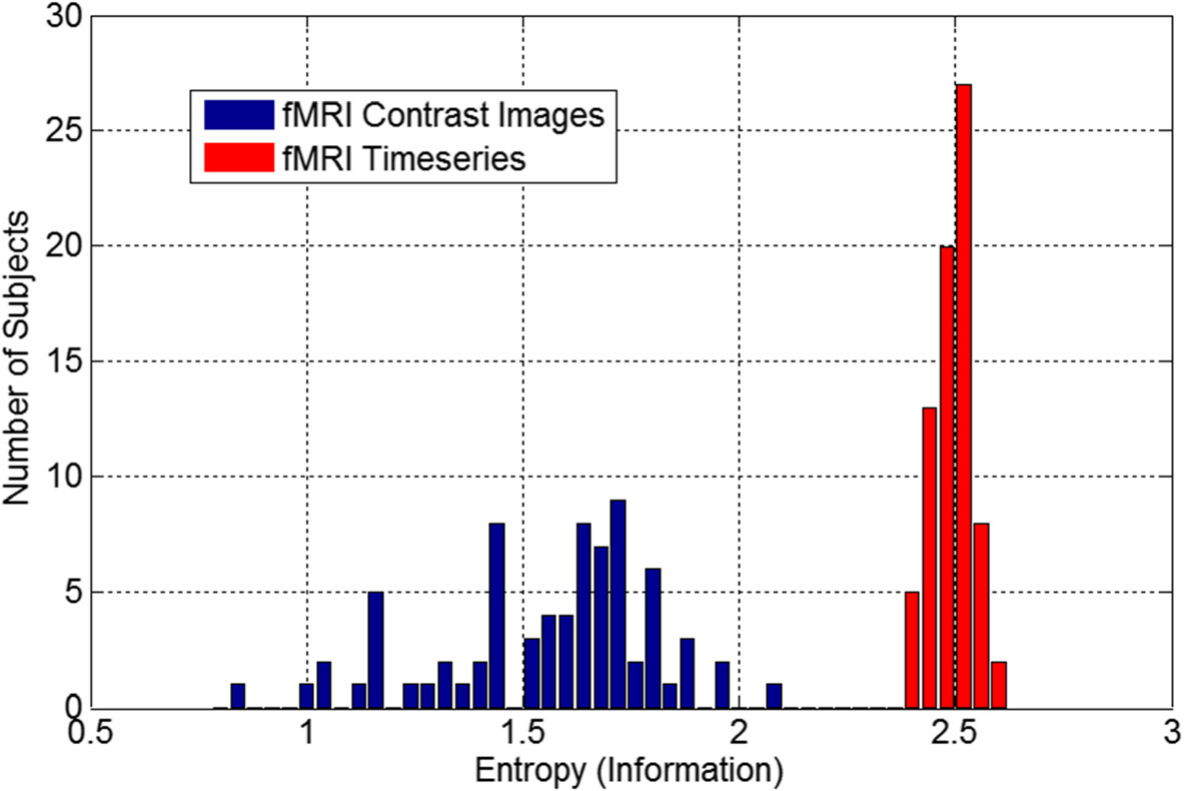
**Entropy of contrast maps versus entropy of preprocessed fMRI data.** It is quite clear that there is considerably more information contained within the preprocessed time series data relative to the contrast maps extracted from the data.

Another domain of neuroimaging data sharing involves data density. One can ‘share’ data by virtue of the tables included in published papers, by sharing result images containing values at all points in the brain, or by sharing the full time-series data. This spectrum roughly maps into the information density of a given data set, and this has implications for its utility. For example, contrast maps specific to a task have been shown to be sensitive to underlying connectivity networks, indeed, applying independent component analysis (ICA) to contrast maps from a task-based study reveals networks of regions showing common across-subject covariation, which resemble with widely studied resting fMRI networks [33]. This is likely due to a ‘fortuitous’ biasing of the task-based activity by the underlying connectivity. However sharing only contrast images comes at a significant cost; that is loss of information. As shown in [33], though it is clear that one can estimate similar networks from second-level data, the estimated networks are noisier than those estimated from raw data, and thus more subjects would be needed to compensate for this. One can directly estimate the amount of information in contrast images versus raw data using entropy. Figure 4 shows an example of the average entropy calculated from the contrast images of 20 subjects (blue), as well as the average entropy calculated from the raw data (red); it is obvious that the variability among subjects is much higher and the entropy is much lower for the contrast images. In addition, there is information in the time-series data that are not visible from the average maps, for example without the raw data one is unable to make inferences about the dynamics of the network patterns (i.e., the chronnectome) [34], a rapidly growing area of fMRI investigation. In addition, data fusion approaches can benefit greatly from additional information about each modality [35,36].

#### Concern: Privacy

It is obvious that if maximizing information is the only goal then sharing of raw data should always be done. However in some cases there are goals which compete against the maximization of information, such as the need to preserve privacy. In some cases privacy is of paramount importance and can be a major barrier to data sharing. High dimensional datasets entail a high risk for re-identification despite meeting current privacy standards (e.g., HIPAA) –a common concern in the context of high dimensional biological datasets (e.g., genetics, MRI images). The recent Netflix competition highlighted concerns about phenotypic data when some competitors inadvertently re-identified individuals from anonymous datasets [37] (http://www.netflixprize.com; http://www.wikipedia.org/wiki/Netflix_Prize). The well-known example of genetic *reidentification* from datasets anonymized per National Institutes of Health (NIH) guidelines is another cautionary tale [38-41].

Data usage agreements (DUA) are a potential solution for enabling access to data while maintaining participant privacy, but unfortunately they have significant limitations for large studies, for example getting approval for many DUAs, each of which may require institutional approach, can be cumbersome and slow. NIH’s centralized database efforts, such as the National Database for Autism Research (NDAR) [42], are a step forward, but are US-based and require a federal-wide assurance number (FWA), limiting the international sharing of data, and still requires centralized downloading and manual organization of all data. The incorporation of a DUA management tool is one possibility which would be extremely helpful for building large consortia.

Data sharing efforts like ADNI [43], HCP [44], INDI [8], and openfMRI [45] are open, provide deidentified data, and use the DUA approach. Other approaches, in particular ENIGMA [46], which work with more sensitive genetic data, do not require data to be shared, but instead work with individual investigators to have them run scripts on their data to provide desired summary measures for meta-analysis. This is more private, though not in a quantifiable way.

**Figure 5:**
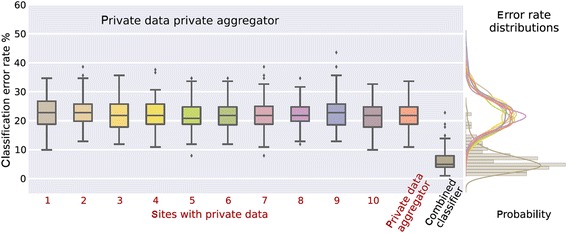
**Classification results on private data.** Differentially private approach to data sharing which enables the use of a support vector machine classifier on data from multiple privacy sites to be pooled together, resulting in a significantly decreased error rate. Notably, with enough sites, the error rate is comparable to that one would obtain if the data were completely open [47].

Another solution to address the above concerns about privacy is to provide tools to share data in a way that protects privacy while still enabling maximal information extraction via analytic approaches, such as multivariate classification [17,47]. Systems that attempt privacy-preserving computation fall into three categories. The first set provides *plausible privacy* by arguing that sharing only data derivatives guarantees privacy since the raw data is not shared (this is the ENIGMA model). Plausibly private systems are best described as not blatantly non-private. A second class of systems, called *definitional privacy,* define privacy via some legal definition of de-anonymization (e.g., the safe harbor clause of HIPAA); by removing certain features*.* Such approaches provide legal or policy guarantees, but make no formal claims of re-identifiability. The final class of systems provide *technological privacy*; privacy is defined as a property of the data (as in k-anonymity [48]) or a property of a data processing algorithm (as in differential privacy [49]). These definitions give an operational meaning to privacy and provide limits on the ability to re-identify an individual. Such systems are not without precedent: in the genetics community, ViPAR [50] and dataSHIELD [51] have used P2P data technologies to support the sharing and aggregate analysis of distributed data, while leaving data control at local sites. Figure 5 provides an example of a differentially private approach to data sharing which results in dramatically improved error rates for a multivariate classifier, the support vector machine, compared to the rates one would get without access to the private data.

The development of privacy preserving analysis approaches is an example of maximizing information while addressing the important concern of privacy. The solution discussed also touches on the use of flexible analytic approaches, such as multivariate classification. Such tools are essential in our quest to make sense of the complex data we are collecting and ultimately, we hope, the human brain. Regarding sharing of raw (and preprocessed data), a recent large consortium (over 5,000 rest fMRI data sets) on reproducibility and replicability (CoRR) of resting fMRI is currently available through COINS and NITRC [52,53]. It will be very interesting to see how this data is used, and certainly it would be possible to systematically compare, and with larger numbers, the various points on the data sharing spectra that we discuss. Though sharing of raw data will always give the most flexibility, there are also great benefits to sharing intermediate data. For example, many interesting findings have emerged in the area of meta-analysis or of the analysis of statistical maps calculated from imaging data [45,54,55].

### Data analysis

**Figure 6:**
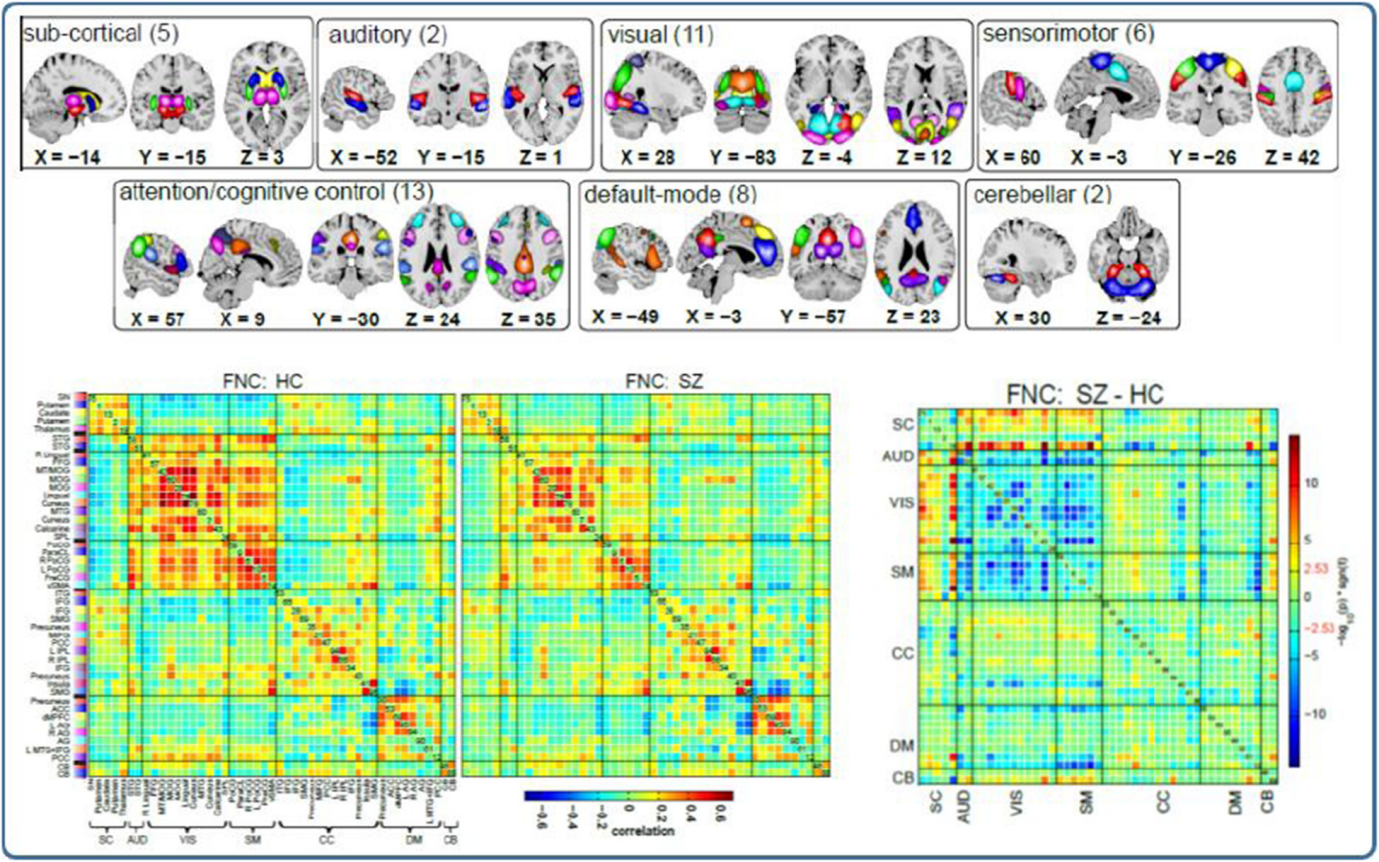
**Example of parcellation using ICA [** 60 **] including component maps (top) separated into categories based on the anatomic location and FNC or among-network connectivity which can be summarized via the cross-correlation among network time courses (bottom).** Results for health individuals (HC), patients with schizophrenia (SZ), and the difference are also shown.

In this final section we touch on the last domain – the analytic approach. There are a wide range of options for analyzing fMRI data ranging, such as approaches which considers only single voxels or regions of interest one-by-one to those that work on the full data set at once in a multivariate framework. While it is not possible to do justice to the breadth of approaches currently available, one main emphasis in more recent years has been a focus on networks [56] rather than individual regions or voxels. Such approaches, including whole-brain seed-based to ICA-based approaches, enable beautiful parcellations of brain function to be estimated from the data while also enabling statistical comparisons of the connectivity both within and among networks (the latter is called functional network connectivity or FNC [57,58]). Figure 6 (top) shows an example of a group ICA-based [59] parcellation and also an example of the FNC, or among-network connectivity (bottom) both within healthy individuals (bottom left), schizophrenia patients (bottom middle) and differences (bottom right). While possible on summary maps as described earlier [33], the use of such approaches is not optimal without access to the original data.

**Figure 7:**
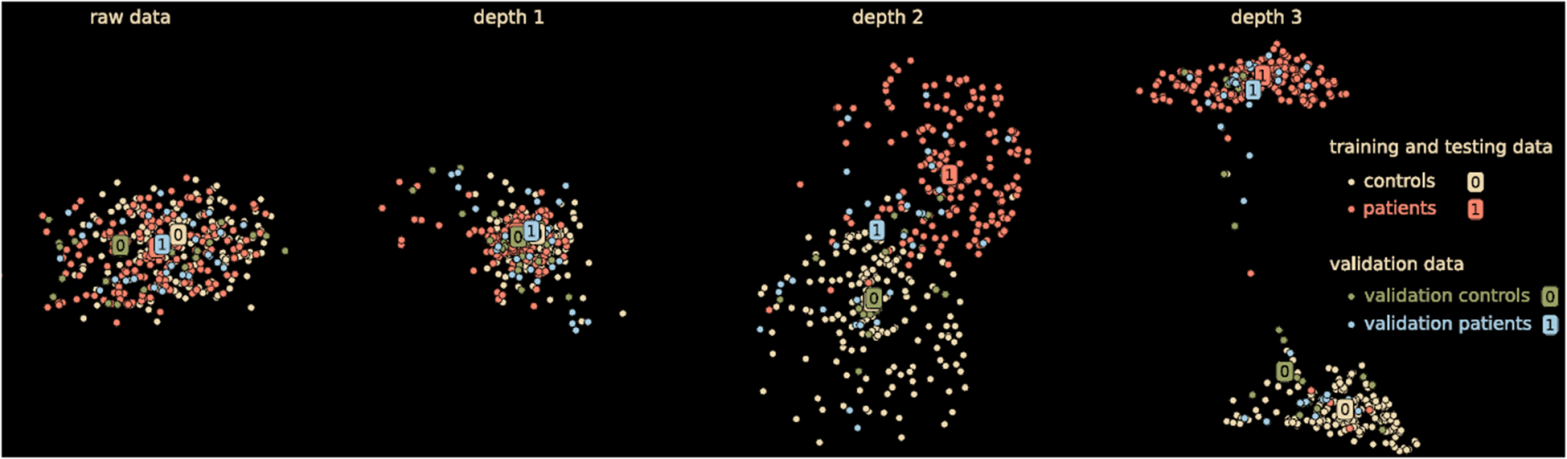
**Impact of depth of model on classification accuracy in brain imaging data.** As the depth of the learner increases (from left to right) the discriminative power of the learnt features increases as well. Notably, the subjects that were held out are also well discriminated, meaning that deep learning generalizes to unseen data. The mapping facilitates analysis of large datasets by displaying complete data in a single figure in a way that highlight data regularities [61].

Another example of a ‘high information’ analysis approach is the use of multivariate classification. One recent approach that has shown promise for neuroimaging data is deep learning [61,62], a technique which has performed quite well in the area of social network mining, image processing, and digit recognition among others. The idea is to learn hidden, possibly nonlinear, aspects of data which in the end can significantly improve classification performance. Figure 7 shows an example of the impact of model depth on the results from a brain imaging analysis. It is encouraging to see that in a cross-validated approach the groups appear to be better separated with increasing depth. This is of course no guarantee that deep learning will work in all cases, but it does suggest there is potential for learning important information from brain imaging data which might not be immediately obvious from a simple group difference.

#### Concern: Interpretability

One key concern with the use of more complex analytic approaches is the potential for overfitting the data as well as the lack of interpretability, especially with nonlinear approaches. These are valid concerns, the first can be addressed by using best practices in cross-validation of results (e.g., k-fold cross-validation) and careful evaluation of potential confounding variables. The latter represents a desire to interpret the results. Approaches like ICA are quite often linear, and can thus be quite readily interpreted, and the most widely-used ICA approaches optimize for both independent and sparsity measures with considerable success [63]. Fortunately, even for more complex methods, there are ways to project the data into a domain that can be interpreted. This however has not been a major goal of the initial wave of results, which primarily focus on classification performance. But even highly nonlinear approaches, such as deep learning, can be carefully evaluated at each of the layers to interpret the underlying results. However, much more work is needed in this area.

In summary, flexible data analysis approaches can be highly informative especially when the underlying signals of interest are complex and poorly understood. Ultimately, there is a trade-off in the use of a simpler model with fewer parameters; however, a simpler model does not guarantee a better solution. One example summarized in [56] shows, in the case of predicting age, the mean activity across the entire brain gives better predictive power over more complex approaches, yet when predicting diagnosis, a connectivity-based measure was more informative than the simpler measures. That being said, given the high complexity of the brain and questions we are asking, and the extremely simple models that are most widely used in brain imaging, there is substantial room for growth in the area of more flexible modeling approaches which will likely lead to an increased understanding of brain structure and function. This has already been born out, for example functional connectivity [34,64,65] which was initially dismissed by much of the field, has grown into a major research focus.

## Conclusions

Data sharing in neuroimaging is alive and well. This review has focused upon the concept of maximization of information, which is extremely important if we are to move our understanding of the brain forward. Consider the fact that we are still finding new information within very complex fMRI data sets that was not initially revealed (such as the recent focus on time-varying connectivity [34]). Current approaches are taking a variety of practical shortcuts to push data sharing forward, such as focusing only on meta-analytic approaches or sharing of only contrast images. While such approaches have their place and are extremely useful, we must not lose sight of the goal of making all collected data available to the community. Within the domains of data capture, data density, and data analysis I have tried to provide some examples, challenges, and solutions in order to foster this ongoing discussion. I look forward to the future and believe the combination of 1) technological advances and tools to assist investigators in collection of high quality data in a way that can be easily shared; 2) approaches to confront storage and computational barriers associated with sharing of the most raw form of the data; and 3) advanced algorithms to enable data-mining of rich data sets even in the context of possible constraints, such as privacy concerns, will move the field ahead at a rapid pace to help fill in the huge gaps in knowledge we have about human brain function and ultimately may help improve the lives of those with devastating brain disease.

## Abbreviations

COINS: Collaborative informatics and neuroimaging suite
DUA: Data usage agreement
fMRI: Functional magnetic resonance imaging
oCOINS: Offline COINS
FNC: Functional network connectivity
HC: Healthy control
ICA: Independent component analysis
NDAR: National database for autism research
NIH: National Institutes of Health
SZ: Schizophrenia patient
